# Antibody Response and Disease Severity in Healthcare Worker MERS Survivors

**DOI:** 10.3201/eid2206.160010

**Published:** 2016-06

**Authors:** Abeer N. Alshukairi, Imran Khalid, Waleed A. Ahmed, Ashraf M. Dada, Daniyah T. Bayumi, Laut S. Malic, Sahar Althawadi, Kim Ignacio, Hanadi S. Alsalmi, Hail M. Al-Abdely, Ghassan Y. Wali, Ismael A. Qushmaq, Basem M. Alraddadi, Stanley Perlman

**Affiliations:** King Faisal Specialist Hospital and Research Center, Jeddah, Saudi Arabia (A.N. Alshukairi, I. Khalid, W.A. Ahmed, A.M. Dada, D.T. Bayumi, L.S. Malic, H.S. Alsalmi, G.Y. Wali, I.A. Qushmaq, B.M. Alraddadi);; King Faisal Specialist Hospital and Research Center, Riyadh, Saudi Arabia (S. Althawadi, K. Ignacio);; General Directorate of Infection Prevention and Control, Ministry of Health, Riyadh (H.M. Al-Abdely);; University of Iowa Department of Microbiology, Iowa City, Iowa, USA (S. Perlman)

**Keywords:** antibody, Middle East respiratory syndrome coronavirus, MERS-CoV, ELISA, indirect immunofluorescence assay, IFA, healthcare workers, immune response, viruses, Jeddah, Saudi Arabia

## Abstract

We studied antibody response in 9 healthcare workers in Jeddah, Saudi Arabia, who survived Middle East respiratory syndrome, by using serial ELISA and indirect immunofluorescence assay testing. Among patients who had experienced severe pneumonia, antibody was detected for >18 months after infection. Antibody longevity was more variable in patients who had experienced milder disease.

A study evaluating the immune response in patients infected with severe acute respiratory syndrome coronavirus (SARS-CoV) showed antivirus antibodies in survivors can be detected by ELISA and immunofluorescence assay (IFA) for up to 24 months after infection (*1*). Another study revealed that SARS-CoV antibodies were not detectable at 6 years after infection (*2*). Antibody response to Middle East respiratory syndrome coronavirus (MERS-CoV) typically is detected in the second and third week after the onset of the infection (*3*–*5*), but little is known about the longevity of the response or whether the decrease in antibody response over time correlates with the severity of the initial infection. We conducted a longitudinal study of antibody response among a cohort of MERS survivors who had been treated at King Faisal Specialist Hospital and Research Center in Jeddah, Saudi Arabia (KFSHRC-J).

## The Study

Our research proposal was approved by the KFSHRC-J institutional review board. Written informed consent was obtained from all study participants. During the Jeddah MERS outbreak in 2014, we tested specimens from 1,412 patients with suspected MERS-CoV infection by using a real-time reverse transcription PCR (rRT-PCR) assay. We identified 40 confirmed cases on the basis of rRT-PCR–positive specimens obtained by nasopharyngeal swab or bronchoalveolar lavage, as described previously (*6*; Technical Appendix). For each patient, >2 specimens were analyzed and rRT-PCR was conducted twice. Eighteen of 40 cases were in healthcare workers (HCWs); 12 of these 18 HCWs were symptomatic. The 6 asymptomatic HCWs were identified through contact tracing during active hospital surveillance for MERS cases. The patient cohort for this study consisted of 9 HCWs who were MERS-CoV–positive on the basis of rRT-PCR results and who agreed to provide blood samples for serial serologic testing for MERS-CoV by ELISA and IFA. 

Patients’ medical records were reviewed for information on demographic characteristics, comorbidities, clinical presentation, intensive care unit admission, and outcome. Patients were classified into 4 categories according to their clinical presentation: asymptomatic, upper respiratory tract infection, pneumonia, or severe pneumonia. Patients with severe pneumonia were those who required intubation and ventilatory support and were treated in an intensive care unit. Serial ELISA and IFA testing was performed at 3, 10 and 18 months after illness onset (Technical Appendix). Specimens were considered to represent previous infection only when ELISA and IFA test results both were positive. Microneutralization testing was not available in the KFSHRC-J laboratory.

Disease onset corresponded to the date of the first MERS-CoV–positive rRT-PCR result. Data were available for analysis from 9 patients who were MERS-CoV–positive and had serial MERS-CoV serologic testing at 3 and 10 months after illness onset. Patients with severe pneumonia who were MERS-CoV-antibody–positive at 10 months had follow-up testing at 18 months. Serum samples could not be obtained from patient 3, who was also MERS-CoV-antibody–positive at 10 months. All patients were initially healthy without underlying conditions except patient 2 (Table), who had hypothyroidism. Four of the 9 patients were women; 2 of them, patients 2 and 8, were 32 weeks and 20 weeks’ pregnant, respectively, when they had MERS-CoV infection. Average patient age was 38 years (range 27–54 years). 

**Table:** Antibody response in 9 confirmed survivors of MERS-CoV infection, by selected demographic and clinical characteristics, King Faisal Specialist Hospital and Research Center, Jeddah, Saudi Arabia, 2014*

Patient no.	Age, y/sex	Clinical presentation	Test results										
			PCR, C_t_			Serology at 3 mo			Serology at 10 mo			Serology at 18 mo	
			NPS	BAL		ELlSA†	IFA‡		ELISA†	IFA‡		ELISA†	IFA‡
1	49/M	Severe pneumonia	28	26		+ (3.17)	+		+ (2.99)	+		+ (3.3)	+
2	33/F	Severe pneumonia	31	26		+ (2.7)	+		+ (2.09)	+		+ (2.9)	+
3	54/F	Pneumonia	34	ND		+ (2.91)	+		+ (1.9)	+		ND	ND
4	40/M	Pneumonia	32	ND		+ (1.29)	+		– (0.65)	–		ND	ND
5	37/M	Pneumonia	35	ND		+ (3.2)	+		+ (1.2)	–		ND	ND
6	36/M	URTI	32	ND		– (0.07)	–		– (0.07)	–		ND	ND
7	27/F	Asymptomatic	33	ND		– (0.046)	–		– (0.04)	–		ND	ND
8	28/F	Asymptomatic	32	ND		– (0.12)	–		– (0.06)	–		ND	ND
9	35/M	Asymptomatic	33	ND		– (0.07)	–		– (0.04)	–		ND	ND


*+, positive; –, negative; BAL, broncholaveolar lavage; Ct, cycle threshold; IFA, indirect-immunofluorescence assay; MERS-CoV, Middle East respiratory syndrome coronavirus; ND, not done; NPS, nasopharyngeal swab; URTI, upper respiratory tract infection. †ELISA for MERS-CoV S gene antibody; positive defined as a value >1.1, negative as <0.8, and borderline as between 0.8 and 1.1. ‡IFA for MERS-CoV IgG; endpoint titers not done.

Of the 9 patients, 2 had severe pneumonia, 3 had milder pneumonia not requiring intensive care, 1 had upper respiratory tract disease, and 3 remained asymptomatic. All patients recovered without sequelae. The 2 patients with severe pneumonia had the highest antibody titers detected among all patients and remained MERS-CoV-antibody–positive when tested at 18 months after illness onset. They also had prolonged viral shedding documented by persistent positive rRT-PCR results for 13 days (patient 1) and 12 days (patient 2); rRT-PCR analyses were negative after 2–5 days for patients 4–9. rRT-PCR was only repeated at day 13 for patient 3, and the result was negative. Three patients with pneumonia were MERS-CoV-antibody–positive at 3 months, but antibody was detected in only 1 of the 3 at 10 months (Table). All patients who had an upper respiratory tract function or remained asymptomatic had no detectable antibody response on the basis of ELISA and IFA results.

## Conclusions

Our results indicate that the longevity of the MERS-CoV antibody response correlated with disease severity. Accordingly, 2 patients with severe MERS-associated pneumonia had a persistent antibody response detected for >18 months after infection, whereas patients with disease confined to the upper respiratory tract or who had no clinical signs had no detectable MERS-CoV antibody response. Two previous studies have described longitudinal analyses of MERS-CoV surface glycoprotein–specific antibody responses in recovered patients. In the first study, which described a MERS outbreak in Jordan (*7*), MERS-CoV antibodies, including neutralizing antibodies, were still detectable in 7 patients with pneumonia 13 months after infection; most of these patients had severe pneumonia. In the second study, Drosten et al. (*8*) demonstrated that MERS-CoV neutralizing antibodies were produced at low levels after mild or subclinical infection and were potentially short-lived.

The results of our study have implications for understanding the pathogenesis and the treatment of MERS. First, patients with mild or subclinical infections who had no detectable antibody response might be at risk for recurrent infection and would also not be detected in population-based studies, resulting in falsely low prevalence rates. Previous studies suggest that neutralizing antibodies were not sufficient to clear MERS-CoV, because neutralizing antibodies were detected in up to 50% of fatal MERS cases and antibody levels did not correlate well with virus load in the lungs (*3*). T-cell responses are critical for protection from subsequent challenge in animals experimentally infected with SARS-CoV (*9*). Although T cells have persisted up to 6 years in SARS survivors (*2*), whether these patients would have been protected if infected a second time with SARS-CoV is unknown. Additional studies will be required to assess the relative importance of T- and B-cell responses in MERS survivors. Second, we speculate that patients with low-level virus replication could provide a reservoir for infection of highly susceptible humans (i.e., those with underlying conditions). Such patients would be difficult to detect because they are only transiently positive for MERS-CoV antibody or might never mount an antibody response to MERS-CoV. Third, patients who recovered from severe pneumonia associated with MERS probably would be good candidates for providing MERS-CoV–specific convalescent-phase serum samples for use in treatment trials.

Our study is limited by the small number of patient numbers and the lack of neutralizing antibody testing. ELISA is highly sensitive but might cross-react with seasonal human coronavirus antibodies (*10*); it is useful as a screening test because it is 10-fold more sensitive than IFA. An IFA is required for confirmation (*11*), and use of a spike protein–specific IFA greatly diminishes the likelihood of cross-reactivity. Neutralization assays are considered definitive and must be performed whenever the results of ELISA and IFA are not conclusive (*12*). In the 9 patients reported here, ELISA and IFA results were consistent and conclusive. A limitation of this study is that serologic testing from single patients was not performed on the same day; therefore, only qualitative but not quantitative conclusions about changes with time after illness onset can be made. 

In conclusion, our results indicate that MERS-CoV antibody persistence depends on disease severity. Further studies are required to determine the role of the virus-specific T-cell response in MERS patients and determine whether patients with mild infections are at risk for reinfection and would therefore benefit from vaccination. Our data also show that potential donors of MERS-CoV convalescent-phase serum samples are limited to patients who recover from severe pneumonia.

Technical Appendix Methods and materials used for a longitudinal study of antivirus antibody response after MERS-CoV infection, conducted at King Faisal Specialist Hospital and Research Center, Jeddah, Saudi Arabia, 2014.
