# Supplementary material for: Antibody Response and Disease Severity in Healthcare Worker MERS Survivors
Source: Emerg Infect Dis. 2016 Jun;22(6):1113–5. doi: 10.3201/eid2206.160010 (PMC4880093; doi:10.3201/eid2206.160010)
Supplement: Technical Appendix — Methods and materials used for a longitudinal study of antivirus antibody response after MERS-CoV infection, conducted at King Faisal Specialist Hospital and Research Center, Jeddah, Saudi Arabia, 2014. [file 16-0010-Techapp-s1.pdf]

Article DOI: http://dx.doi.org/10.3201/eid2206.160010

Antibody Responses and Disease Severity in Healthcare Worker MERS Survivors

Technical Appendix

Methods and Materials

Quantitative Real-Time Reverse Transcription PCR (rRT-PCR)

MERS-CoV rRT-PCR was performed as described previously (*1*) using RNA extracted from nasopharyngeal swabs and/or bronchoalveolar lavages obtained from patients in April 2014. Briefly, nasopharyngeal and BAL samples were immediately transported on ice to the lab. Nucleic acid extraction was performed using an EZ1 advance XL Extraction System and an EZ1 Virus mini kit v2.0 (Qiagen, Germany). The EZ1 kit provides a fully automated procedure for purification of viral nucleic acid. cDNA was prepared and analyzed by PCR using a Light Cycler (Roche, Switzerland), with a Realtime Ready RNA Virus Master (Roche) and primers complementary to the region upstream of the E-gene and to the Orf1a gene (TIB MOLBIOL, Germany). Negative and positive controls were included in each run. Positive controls produced exponential curves with well-defined logarithmic, liner and plateau phases, documenting the specific amplification of MERS-CoV RNA. Negative controls produced no PCR signal. The data were interpreted independently by two different technologists with final approval by an experienced molecular virologist. The C_t value cutoff was 35.

ELISA

ELISAs were performed as described previously (*2*,*3*). Briefly, a MERS-CoV-specific ELISA kit was purchased from EUROIMMUN, Germany. The assay uses microplates coated with purified recombinant MERS-CoV S1 spike protein. Use of the S1 fragment, the least conserved protein in the CoV genome, as antigen reduces the risk of cross reactivity with antibodies against other human coronaviruses. Positive and negative controls, included in the kit, were used according to the manufacturer's instructions. Samples with an optical density equal to or more than 1.1 were considered as positive and those with less than 0.8 as negative. Results

between 0.8 and 1.1 were considered borderline. Serial dilution and triplicate runs were not required according to the manufacturer's instructions. 1:101 dilutions were used.

Anti-MERS Coronavirus IIFT (IgG)

The Anti-MERS-CoV IIFT (IgG) kit is a validated indirect immunofluorescence assay (EUROIMMUN, Germany) that is used for qualitative detection of IgG antibody to MERS-CoV (*4*). The kit was used as per the manufacturer's instructions. Positive and negative controls were run with all samples. Staining with anti-MERS-CoV antibodies results in a distinctive pattern of fluorescence in the cytoplasm of infected cells, consisting of fine to coarse granular structures. Samples are reported as positive according to the degree of fluorescence observed when compared to positive and negative controls. Reactions are considered positive when fluorescence is observed in majority of the cells (50–100 cells per low power field).The antigen is coated on slides with 2 Biochips (one Biochip coated with MERS-CoV infected cells (species EU 14) and the other Biochip with noninfected cells (not specified in the instruction leaflet). MERS-CoV-specific antibody is detected using Fluorescein-conjugated goat anti-human IgG. The positive controls are anti-mitochondrial antibodies (AMA, IgG human). Negative controls antibody were provided by the manufacturer and consisted of human sera known to be negative for antibody reactive with MERS coronavirus and SARS coronavirus. The recommended sample dilution for qualitative evaluation was 1:100 in sample buffer. Only one dilution was used as per instructions. Stained slides were examined using a Zeiss Axioskop2 Plus fluorescent microscope with a wavelength filter of 490nm. The slides were examined using a low power objective (x20). Several fields were examined for each sample.

The following subjective scoring was applied:

1. Negative - No fluorescence or 1+ intensity (dim or dull apple-green fluorescence, lacking in sharpness, no focal labeling)

2. Weakly positive - 2+ intensity (clear distinguishable apple-green focal fluorescence)

3. Moderately positive - 3+ intensity (bright focal apple-green fluorescence)

4. Strongly positive - 4+ intensity (very bright focal apple-green fluorescence)

The positive control contained numerous cells that fluoresced strongly (4+). There were at least 50 –100 cells noted per low power field. The Biochip slides are also coated with numerous cells (50–100 per LPF). As noted, in the positive samples, numerous cells showed 3+ to 4+ fluorescence. There were at least 20–30 positive cells.

There are five wells per slide. In each well, there are two biochips, one biochip contains infected MERS-coronavirus Cells and the other biochip contains noninfected cells. The positive control is added to the first well and the negative control is added to the second well. Patient samples are added to the remaining three wells. If the sample is positive, a reaction will be observed in the biochip that contains the MERS-coronavirus infected cells, whereas the biochip with noninfected cells will show no fluorescence or very dim, nonfocal fluorescence.

References

1. Corman VM, Müller MA, Costabel U, Timm J, Binger T, Meyer B, et al. Assays for laboratory confirmation of novel human coronavirus (hCoV-EMC) infections. Euro Surveill. 2012;17:20334. PubMed

2. Drosten C, Meyer B, Müller MA, Corman VM, Al-Masri M, Hossain R, et al. Transmission of MERS-coronavirus in household contacts. N Engl J Med. 2014;371:828–35. PubMed http://dx.doi.org/10.1056/NEJMoa1405858

3. Aburizaiza AS, Mattes FM, Azhar EI, Hassan AM, Memish ZA, Muth D, et al. Investigation of anti-middle East respiratory syndrome antibodies in blood donors and slaughterhouse workers in Jeddah and Makkah, Saudi Arabia, fall 2012. J Infect Dis. 2014;209:243–6. PubMed http://dx.doi.org/10.1093/infdis/jit589

4. Buchholz U, Müller MA, Nitsche A, Sanewski A, Wevering N, Bauer-Balci T, et al. Contact investigation of a case of human novel coronavirus infection treated in a German hospital, October–November 2012. Euro Surveill. 2013;18:20406. PubMed
